# The Role of Malocclusion and Oral Parafunctions in Predicting Signs and Symptoms of Temporomandibular Disorders—A Cross-Sectional Study

**DOI:** 10.3390/dj12070213

**Published:** 2024-07-10

**Authors:** Luka Šimunović, Marina Lapter Varga, Dubravka Negovetić Vranić, Ivana Čuković-Bagić, Lana Bergman, Senka Meštrović

**Affiliations:** 1 Department of Orthodontics, School of Dental Medicine, University of Zagreb, 10000 Zagreb, Croatia; lsimunovic@sfzg.hr (L.Š.); lapter@sfzg.hr (M.L.V.); 2 Department of Pediatric and Preventive Dentistry, School of Dental Medicine, University of Zagreb, 10000 Zagreb, Croatia; dnegovetic@sfzg.hr (D.N.V.); bagic@sfzg.hr (I.Č.-B.); 3 Department of Prosthodontics, School of Dental Medicine, University of Zagreb, 10000 Zagreb, Croatia; bergman@sfzg.hr

**Keywords:** temporomandibular disorders, malocclusion, parafunctions, children

## Abstract

Background: The aim of this study was to examine to what extent malocclusion and parafunctional habits contribute to the development of signs and symptoms associated with temporomandibular disorders (TMD) in schoolchildren with mixed dentition in Croatia in a sample of 338 children, aged 9 to 15 years. Methods: TMD signs and symptoms assessed by the clinician were joint function and pain, masticatory muscles tenderness, range of mandibular motion, and joint sounds. To evaluate subjective symptoms and parafunctions, children and parents were asked about the presence of headaches, jaw locking, temporomandibular joint (TMJ) sounds, pain during mouth opening, or bruxism, as well as parafunctions like biting pencils or nails, chewing hard candies or ice, daily gum chewing, opening bottles with teeth, engaging in jaw play, thumb-sucking, and clenching/grinding teeth. Results: At least one symptom of a TMD was pronounced in 142 participants (42.0%). The most commonly reported parafunction was pencil or nail biting, present in 25.1% of participants. Class II malocclusion increased the likelihood by 2.6 times, pencil or nail biting by 2.34 times, and clenching/grinding teeth by 8.9 times that the subject would exhibit at least one TMD symptom. Conclusions: Every child with mixed dentition should undergo a brief examination of the TMJ, especially in cases of Class II malocclusion, pencil or nail biting, and teeth clenching or grinding, as these have all been identified as significant risk factors that increase the likelihood of experiencing TMD symptoms. This highlights the need for proactive screening and assessment by healthcare providers to reduce the risk and prevalence of TMDs in affected children and ensure timely diagnosis and treatment.

## 1. Introduction

### 1.1. Definition, Historical Context, and Evolution of Treatment Approaches

Temporomandibular disorders (TMDs) encompass a range of clinical signs and symptoms characterized by pain and disruptions in the functioning and structure of the masticatory system, particularly the temporomandibular joint (TMJ) and the masticatory muscles (MMs) [1,2]. According to the American Association of Dental Research, a TMD refers to musculoskeletal and neuromuscular conditions affecting the TMJ, MM, and related tissues [3]. A TMD is one of the most prevalent sources of non-dental facial pain [4,5,6]. Managing TMDs has been debated for nearly a century, with significant historical milestones shaping current understanding. In 1934, Costen linked TMJ issues and ear pain to changes in the vertical dimension. Later, the Gnathological society emphasized jaw functioning and occlusal positions, advocating for a canine-protected occlusion and eliminating centric relation–centric occlusion (CR-CO) discrepancies [4]. Since then, theories have evolved, with recent clinical trials favoring a biopsychological model over mechanical approaches for managing TMDs, highlighting the efficacy of placebo medications, deprogramming appliance therapy, and occlusal equilibration [4,7,8].

### 1.2. Temporomandibular Disorders in Children and Adolescents: Signs, Symptoms, and Challenges

Functional disturbances within the orofacial system can be present in children with primary dentition but are often underdiagnosed. These issues can progress into TMDs as children grow [9]. Common clinical signs in children and adolescents include tenderness in the MM and TMJ, joint noises, and headaches [10]. TMJ sounds, pain, and limited mouth opening are primary symptoms, with TMJ clicking often used for the initial screening of intra-articular TMJ disorders [11,12,13,14]. However, disc displacement with a reduction without a limited opening typically does not require immediate treatment [11,12], and some researchers consider TMJ clicking a normal condition [12]. Longitudinal studies show that TMD signs and symptoms in children fluctuate, with some improving and others worsening over time [10,15]. Severe signs are uncommon, and few children require functional treatment [10]. The etiology of TMDs is complex, involving trauma, parafunctional habits, and anatomical, pathological, and psychosocial factors [16,17,18]. Proposed causes include unstable occlusion (insufficient uniform contact among teeth during occlusal closure), joint hypermobility, psychological factors (e.g., anxiety and depressive thoughts), and genetic predisposition [19,20]. The connection between TMDs and malocclusions or occlusal interferences has been extensively researched, with certain malocclusions like Angle Class II and III, significant overjet, dental crowding, anterior open bite, and posterior crossbite more strongly linked to TMD development [15,17,21,22]. These associations may result from unstable occlusion and weaker muscles [16,17]. Reduced occlusal support impacts muscle activity, bite force, and jaw movements [23], although premature loss of primary teeth does not seem to contribute to TMDs [16]. Parafunctional habits, especially bruxism, are significantly linked to TMDs in children and adolescents [21,24]. The prevalence of TMD signs and symptoms has increased, partly due to underdiagnosis in children [25,26]. Gender differences in TMD occurrence and presentation are noted in adults, with TMD prevalence in children and adolescents ranging from 20% to 60% [20]. Despite many reports on TMDs in these age groups, effective pain management remains a challenge [27]. Studies suggest that genetic and psychosocial factors, as well as muscle-related strain, play roles in TMDs, but the connection between malocclusion and TMDs in children is unclear [4,28].

Children often struggle to verbalize facial pain and jaw dysfunction, leading to non-definitive histories and underdiagnosis. This emphasizes the need for dentists to be vigilant about early TMJ disorder signs during examinations [29]. Many children experience TMJ symptoms like joint sounds and restricted mouth opening that are not reported or recognized [30].

Therefore, this study aims to examine the extent to which malocclusion and parafunctional habits contribute to the development of TMD signs and symptoms in schoolchildren with mixed dentition in Croatia.

## 2. Materials and Methods

### 2.1. Study Design

A total of 338 children (168 boys and 170 girls) with mixed dentition, ranging in age from 9 to 15 years, were selected from each consecutive patient referred for orthodontic assessment at the School of Dental Medicine, University of Zagreb from January 2022 to January 2023 and were addressed to this cross-sectional study. The design adhered to the STROBE guidelines for cross-sectional studies. All patients underwent examination by the same orthodontist, a tenured full professor in the Department of Orthodontics, who was blinded to the patients’ malocclusions and parafunctional habits to reduce potential bias. Exclusion criteria included children with clefts, syndromes, systemic illnesses, other orofacial pain disorders, visible TMJ deformities on orthopantomogram, prior orthodontic treatment, and those younger than 9 years or older than 15 years. The subjects were categorized into three age groups: 9–10 years, 11–13 years, and 14–15 years. Informed consent for participation in this study was obtained from the subjects’ parents or guardians, and the protocol was approved by the Ethical Committee of the School of Dental Medicine (Class: 8.1-22/115-2; Number: 02/013-AG). The subjects were further divided into groups based on their sex and chronological age.

The examination was conducted using artificial lighting, mouth mirrors, and palpation. All data were meticulously recorded on a designated examination form created for this study. Malocclusions were classified according to the Angle classification system, distinguishing between normal occlusion and malocclusion classes I, II, and III.

The extraoral examination encompassed an evaluation of TMJ function and pain, tenderness in the masticatory muscles, and the range of mandibular motion. The presence of TMJ sounds was assessed by palpating both TMJs with the middle and second fingers during mouth opening, and the results were categorized as palpable clicking, audible clicking, or no signs. Locking and luxation were also observed during mandibular movements. For assessing TMJ pain, both TMJs were palpated bilaterally through the external auditory meati, and the pain was recorded as either unilateral or bilateral, palpable, or related to the palpebral reflex. Muscle pain and tenderness were evaluated through palpation of the temporal muscles, masseter muscles, medial pterygoid muscles, and lateral pterygoid muscles. The mandibular deflection was noted as positive if the mandibular midline deviated to the left or right during opening. The presence of luxation or jaw locking was assessed during mandibular movements. All aforementioned signs and symptoms were then classified as positive or negative. To assess symptoms and parafunctions, the child and the parent or caregiver were asked the following question: “Have you noticed any pain or discomfort in the TMJ region (with an explanation of where this region is for the child), frequent headaches, or audible clicking of the TMJ in the last six months?”. Additionally, they were asked about parafunctional habits such as biting pencils or nails, chewing hard candies or ice, daily gum chewing, opening bottles with teeth, engaging in jaw play, thumb-sucking, and clenching or grinding teeth. Tooth wear was documented according to the Tooth Wear Evaluation System (TWES 2.0) [31].

### 2.2. Sample Size

In our study, the sample size determination was informed by an initial pilot study involving 50 patients, followed by an a priori power analysis, employing a large sample z-test for logistic regression as outlined by Demidenko [32] with variance correction. This analysis aimed to detect significant effects with a two-tailed test at an α error probability of 0.05 and a power of 0.8. Key parameters included an odds ratio of 1.9, a baseline probability (Pr(Y = 1|X = 1) H0) of 0.5, and a binomial distribution for the predictor (X) with a parameter (π) of 0.5. The calculated sample size required was 318 patients, derived to achieve an actual power of 0.80. An additional 20 patients were included, bringing the total to 339 participants, to ensure robustness and reliability in the results.

### 2.3. Statistical Analysis

The data analysis was performed using IBM SPSS Statistics software, version 29.0.1.0 (IBM, New York, NY, USA). We assessed data distribution normality using Shapiro–Wilk and asymmetry tests. The difference in the prevalence of TMD symptoms and parafunctions between gender and age groups as well as occlusions was determined by the chi-square test and its relationship with Cramer’s V test. When the application of the approximation method was inadequate, Fisher’s exact test was used for categorical data. We performed a binary logistic regression analysis to determine the impact of malocclusion and parafunctions as well as gender on TMD symptoms. A *p*-value of 0.05 or less was considered statistically significant.

## 3. Results

The most prevalent age group was 11–12 years, comprising 67.8% of the sample, while the other two age groups were equally represented. The distribution of malocclusion classes based on Angle’s classification was as follows: normal occlusion (13.9%), Class I malocclusion (33.1%), Class II malocclusion (45.9%), and Class III malocclusion (7.1%). The distribution of malocclusion types by age group is presented in Table 1. There was no statistically significant difference in the distribution of malocclusion among sex (*p* = 0.398) and age groups (*p* = 0.133).

At least one symptom of a TMD was pronounced in 142 participants (42.0%). The prevalence of at least one TMD symptom did not show statistically significant differences between sex groups (*p* = 0.898) or among different age groups (*p* = 0.138). The most frequently reported symptoms were clicking during palpation (35.8%), followed by headaches (26.9%) and mandibular deflection (13.0%). Among these, only headaches showed a statistically significant difference between genders, being more commonly reported by girls (15.5% vs. 38.2%, *p* < 0.001). Additionally, headache symptoms were more prevalent in the 11–13 age group compared to the 9–10 group (*p* < 0.001). The most commonly reported parafunction was pencil or nail biting, present in 25.1% of participants. This was followed by chewing gum daily (20.1%), chewing hard candies or ice (16.9%), clenching/grinding (14.8%), opening bottles with teeth (5.9%), jaw play (4.7%), and thumb-sucking (1.8%). Pencil or nail biting (*p* < 0.001), chewing hard candies or ice (*p* = 0.002), chewing gum daily (*p* < 0.001), thumb-sucking (*p* < 0.001), and jaw play (*p* < 0.001) were statistically most frequent in the 9–10 age group. Conversely, opening bottles with teeth was most common in the 11–12 age group (*p* = 0.006). The distribution of parafunctions by sex groups is presented in Table 2.

All TMD symptoms except for locking were mildly associated with pencil or nail biting, with the strongest association being with muscle pain and wear facets. Chewing hard candies or ice was associated with all TMD signs and symptoms, except for clicking during examination and headaches. The strongest relationship was observed with pain in the TMJ area. Daily gum chewing showed a weak association with clicking, locking, and tooth wear. Opening bottles with teeth, thumb-sucking, and jaw play did not show any association with TMD symptoms and therefore were omitted from Table 3. Clenching and grinding were moderately to strongly associated with all symptoms, with the most significant associations being with wear facets, mandibular deflection, and muscle pain (Table 3).

A binary logistic regression (Table 4) was conducted to assess the impact of malocclusion, parafunctions, and sex on the likelihood of participants experiencing TMD symptoms. The model explained 25.2% (Nagelkerke R^2^) of the variance and accurately classified 72.8% of the sample. Among the nine predictor variables, only Class II malocclusion (*p* = 0.020), pencil or nail biting (*p* = 0.005), and clenching/grinding teeth (*p* < 0.001) showed statistical significance (Table 3). Class II malocclusion increased the likelihood by 2.6 times, pencil or nail biting by 2.34 times, and clenching/grinding teeth by 8.9 times (although with a wide confidence interval) that the subject would exhibit at least one TMD symptom.

## 4. Discussion

Chronic pain can significantly impact the daily lives of children and adolescents [33]. Epidemiological data on children with mixed dentition in Croatia are limited, particularly in orthodontics [34,35]. This study provides a foundation for future research in the dental field. Our study identified Class II malocclusion, pencil or nail biting, and clenching/grinding of teeth as significant risk factors for TMD symptoms. Previous studies have reported varying incidence rates for TMD signs and symptoms [36,37,38], with our study finding clicking as the most common clinical sign. Previous research showed clicking rates from 6.8% to 65% [37], attributed to differences in methods and sample sizes. Our manual technique for detecting joint sounds found lower incidences than studies using stethoscopes [36]. This approach aligns with the perspective that joint sounds can be adequately identified through palpation. Using more sensitive detection devices like stethoscopes could potentially lead to increased detection rates and potentially unnecessary and inappropriate treatment [39].

Many authors have reported a higher frequency of TMDs in girls across various age groups [9,10,15,17,22]. However, in contrast to these findings, our study identified only a statistically higher frequency of headaches in girls. No other symptoms showed significant differences between genders. Headaches were observed in 38.2% of female participants, a phenomenon that can be explained by physiological distinctions such as hormonal fluctuations, differences in muscular structure, variations in connective tissue composition, and lower pain threshold levels [40]. Our results also indicate an increase in the prevalence of headaches with age, aligning with findings from a nationwide Austrian study that linked the incidence of headaches to older age in pediatric individuals [41]. A prospective study demonstrated that the presence of other pain conditions, such as headaches, at a baseline serves as a predictive factor for the development of facial pain and TMDs in 11-year-olds [42]. Additionally, there is a bidirectional comorbidity between TMDs and headaches [43]. Therefore, it is advisable to conduct early screenings for signs of TMDs in children and adolescents who experience headaches.

After clicking, the most frequently observed clinical sign was mandibular deflection, present in 13% of cases, which is higher than reported in a study by Farsi et al. [16]. Tuerlings and Limme found mandibular deflection in 19.8% of cases. This variation can be attributed to asymmetrical muscle activity and adaptation to different interferences within the orofacial complex [9]. In our study, we did not differentiate between left or right deflection, although some studies suggest a slight predominance of left deflection [9,16]. The categorization of positive or negative deflection was made at the central point of mouth opening since most mandibles follow S-shaped trajectories to reach the point of maximum mouth opening in the non-deflected position [16].

Numerous reports address the prevalence of oral parafunctions in children [44]. Oral habits like nail biting, teeth clenching, and grinding are as common in children as in adults [45,46,47,48]. Nail biting was the most common oral habit in our study, followed by chewing hard candies or ice and teeth grinding/clenching, with prevalence decreasing with age. Our findings align with similar age group studies [49,50]. While the previous literature does not consistently link bruxism with TMD symptoms, oral parafunctions are important factors in TMD etiology [39]. All TMD symptoms were found to be directly influenced by parafunctions such as pencil or nail biting, chewing hard candies, and teeth grinding. On the other hand, these same parafunctions were also associated with signs of TMDs, except for joint locking and chewing gum. The connection between locking and gum chewing has been recognized in some previous studies [51,52].

Our study suggested that pencil or nail biting increases TMD symptoms by a factor of 2.43, while clenching/grinding increases them by 8.9, though these results should be interpreted cautiously due to wide confidence intervals. Sari et al. [24] reported similar findings with parafunctions linked to TMJ dysfunction. Karibe et al. [53] found habitual clenching significantly correlated with TMD symptoms, suggesting that behavior modification could help. Festa et al. [54] also reported a correlation between awake bruxism and TMDs. In our study, we observed that sleep bruxism was present in approximately 29% of the subjects, with no significant difference between the two groups, while awake bruxism was observed in about 61% of the total subjects.

Our study found that pencil or nail biting is associated with TMD symptoms, particularly wear facets, aligning with research by Farsi et al. [55] who reported similar associations with parafunctional habits. Clenching and grinding are strongly associated with TMD symptoms, corroborated by Fernandes et al. [56]. These findings emphasize the significant role of parafunctional habits in TMD development and the importance of addressing them in clinical settings.

An altered occlusion can disrupt oral function and cause psychosocial issues due to compromised dentofacial aesthetics, with the prevalence of malocclusions in children and adolescents reported to range from 39% to 93% [57]. Several studies have questioned the role of malocclusion in developing TMDs, concluding there is insufficient evidence that malocclusion plays a fundamental role in TMD pathophysiology [4,6,28,58]. However, our findings suggest that Class II malocclusion increases the likelihood of experiencing TMD symptoms by 2.6 times. Further investigation is needed to determine the extent of its contribution [59]. Some clinicians propose that occlusal conditions like deep bites, crossbites, and double bites may predispose individuals to TMDs, with other factors like trauma, emotional stress, bruxism, and systemic conditions also playing a role. The absence of bilateral canine guidance during lateral excursions, particularly in Class II malocclusion, has been considered a significant risk indicator for TMD development, aligning with our study’s findings [60,61]. Additionally, Tecco et al. found that TMD signs and symptoms were 1.6 times more prevalent in individuals with Class II/first division malocclusions [62].

In contrast, a study in 2018 found no association between malocclusion and TMDs [63]. Aboalnaga et al. [59] and Macri et al. [64] found most TMD samples exhibited an Angle Class I molar relationship, with Class II and III less common, while Bilgiç and Gelgor reported a significant association between Class III malocclusion and TMDs [65]. To sum up, signs and symptoms of TMDs in schoolchildren and adolescents can be considered a normal part of growth and development. Therefore, it may not be reasonable to use them as definitive indicators for predicting the future development of TMDs [22]. However, evaluating the TMJ, masticatory muscles, and other aspects of mandibular function during routine dental check-ups for children and adolescents can be valuable in identifying potential risks for TMD development, especially before and after orthodontic therapy.

Despite the competence of healthcare professionals in the treatment of TMDs, patients may receive inadequate treatment or evaluation. This could be due to a lack of awareness about the available therapeutic options and a lack of interdisciplinary collaboration to exchange knowledge and clinical experiences through joint meetings [66,67]. Therefore, it is recommended to establish interdisciplinary clinics involving prosthodontists, oral surgeons, oral medicine specialists, and orthodontists who work together in a coordinated manner, employing various treatment approaches [50,68,69]. Additionally, continuing to develop such programs is crucial to embrace the concept of a multidisciplinary team approach, ultimately improving healthcare services and enhancing the quality of life for these patients.

One limitation of this study is that the signs and symptoms of TMDs can sometimes manifest in individuals without any underlying health issues. While achieving a stable occlusion is a reasonable objective in orthodontic treatment, it is important to note that failing to attain a specific ideal gnathologic occlusion does not necessarily lead to the development of TMD symptoms. Another limitation is the cross-sectional nature of this study, as it only provides a presentation of data collected at a single point in time. Therefore, it cannot establish cause-and-effect relationships or capture changes over time. This makes it challenging to determine the temporal sequence of events or ascertain whether the observed associations are truly causal. Additionally, it is susceptible to recall bias and may not account for long-term trends or fluctuations in the variables being studied. While the binary logistic regression analysis revealed significant associations between Class II malocclusion and parafunctional habits such as nail biting and clenching/grinding teeth with TMD symptoms, the model’s limited explanatory power highlights the need for further research to explore additional factors (such as systemic diseases, e.g., rheumatoid arthritis) and interactions that may contribute to the development of TMD symptoms [70]. Understanding these complexities can lead to more accurate predictive models and better-informed interventions for individuals at risk for TMDs.

## 5. Conclusions

Every child with mixed dentition should undergo a brief examination of the temporomandibular joint, masticatory muscles, and related tissues, especially in cases of Class II malocclusion, pencil or nail biting, and teeth clenching or grinding, as these have all been identified as significant risk factors that increase the likelihood of experiencing TMD symptoms. This highlights the need for proactive screening and assessment by healthcare providers to reduce the risk and prevalence of TMDs in affected children and ensure timely diagnosis and treatment.

## Figures and Tables

**Table 1:** Occlusion distribution according to age groups.

Age Group		Normal Occlusion	Class I	Class II	Class III
9–10	Count	12	20	21	2
	% within Age	21.8%	36.4%	38.2%	3.6%
11–12	Count	30	71	107	21
	% within Age	13.1%	31.0%	46.7%	9.2%
13–15	Count	5	21	27	1
	% within Age	9.3%	38.9%	50.0%	1.9%

**Table 2:** Distribution of parafunctions by sex.

Parafunction	Male	Female	*p* Value
Pencil or nail biting	21.4%	28.8%	
Chewing hard candies or ice	22.0%	11.8%	0.012
Chewing gum every day	23.8%	16.5%	
Opening bottles with teeth	11.9%	0%	<0.001
Thumb-sucking	1.2%	2.4%	
Jaw play	5.4%	4.1%	
Clenching/griding teeth	13.1%	16.5%	

Pearson chi-square/Fisher exact test.

**Table 3:** Relationship between parafunctional habits and TMD signs and symptoms.

		Pencil or Nail Biting	Chewing Hard Candies or Ice	Chewing Gum Every Day	Clenching/Grinding Teeth
Experienced pain in TM region	Yes	60.0%	55.0%	35.0%	75.0%
	No	23.0%	14.5%	19.2%	5.0%
Cramer V		0.201	0.255	0.093	0.572
*p* value		**<0.001**	**<0.001**	0.087	**<0.001**
Reported joint noise (Audible clicking)	Yes	45.0%	30.0%	22.5%	45.0%
	No	22.5%	15.1%	19.8%	4.4%
Cramer V		0.168	0.129	0.022	0.455
*p* value		**0.002**	**0.018**	0.689	**<0.001**
Muscle pain	Yes	66.7%	33.3%	26.7%	73.3%
	No	21.1%	15.3%	19.5%	2.9%
Cramer V		0.299	0.137	0.051	0.694
*p* value		**<0.001**	**0.012**	0.349	**<0.001**
Clicking on examination	Yes	36.4%	21.5%	28.9%	20.7%
	No	18.9%	14.3%	15.2%	2.8%
Cramer V		0.193	0.092	0.164	0.297
*p* value		**<0.001**	0.090	**0.003**	**<0.001**
Locking	Yes	60.0%	60.0%	60.0%	80.0%
	No	24.6%	16.2%	19.5%	8.1%
Cramer V		0.098	0.141	0.122	0.301
*p* value		0.070	**0.009**	**0.025**	**<0.001**
Mandibular deflexion	Yes	54.5%	38.6%	27.3%	63.6%
	No	20.7%	13.6%	19.0%	1.0%
Cramer V		0.262	0.225	0.069	0.730
*p* value		**<0.001**	**<0.001**	0.204	**<0.001**
Headache	Yes	45.1%	18.7%	20.9%	24.2%
	No	17.8%	16.2%	19.8%	3.6%
Cramer V		0.279	0.029	0.012	0.316
*p* value		**<0.001**	0.588	0.832	**<0.001**
Wear facets	Yes	60.0%	40.0%	32.5%	77.5%
	No	20.5%	13.8%	18.5%	0.0%
Cramer V		0.294	0.226	0.113	0.867
*p* value		**<0.001**	**<0.001**	**0.037**	**<0.001**

**Table 4:** Logistic regression model.

	B	Lower and Upper CI	*p* Value
Class II malocclusion	2.61	1.16–5.83	**0.020**
Pencil or nail biting	2.34	1.29–4.25	**0.005**
Clenching/grinding teeth	8.93	3.62–22.00	**<0.001**

B = exponentiated coefficient of a predictor variable (odds ratio); CI = confidence interval.

## Data Availability

The data presented in this study are available on request from the corresponding author.
